# The Interplay of Perceived Risks and Benefits in Deciding to Become Vaccinated against COVID-19 While Pregnant or Breastfeeding: A Cross-Sectional Study in Italy

**DOI:** 10.3390/jcm12103469

**Published:** 2023-05-15

**Authors:** Teresa Gavaruzzi, Marta Caserotti, Roberto Bonaiuti, Paolo Bonanni, Giada Crescioli, Mariarosaria Di Tommaso, Niccolò Lombardi, Lorella Lotto, Claudia Ravaldi, Enrico Rubaltelli, Alessandra Tasso, Alfredo Vannacci, Paolo Girardi

**Affiliations:** 1 Department of Medical and Surgical Sciences, University of Bologna, 40126 Bologna, Italy; 2 Department of Developmental Psychology and Socialization, University of Padova, 35122 Padova, Italy; 3 PeaRL Perinatal Research Laboratory, CiaoLapo Foundation for Perinatal Health, Department of Neurosciences, Psychology, Drug Research and Child Health, University of Firenze, 50139 Firenze, Italy; 4 Department of Health Science, University of Firenze, 50121 Firenze, Italy; 5 Department of Humanities, University of Ferrara, 44121 Ferrara, Italy; 6 Department of Environmental Sciences, Informatics and Statistics, Ca’ Foscari University of Venezia, 30123 Venezia, Italy

**Keywords:** decision making, risk perception, risk/benefit tradeoff, COVID-19 vaccination, pregnancy, breastfeeding, maternal vaccination

## Abstract

The present study examined the role of the perception of risks and benefits for the mother and her babies in deciding about the COVID-19 vaccination. In this cross-sectional study, five hypotheses were tested using data from a convenience sample of Italian pregnant and/or breastfeeding women (N = 1104, July–September 2021). A logistic regression model estimated the influence of the predictors on the reported behavior, and a beta regression model was used to evaluate which factors influenced the willingness to become vaccinated among unvaccinated women. The COVID-19 vaccination overall risks/benefits tradeoff was highly predictive of both behavior and intention. Ceteris paribus, an increase in the perception of risks for the baby weighed more against vaccination than a similar increase in the perception of risks for the mother. Additionally, pregnant women resulted in being less likely (or willing) to be vaccinated in their status than breastfeeding women, but they were equally accepting of vaccination if they were not pregnant. COVID-19 risk perception predicted intention to become vaccinated, but not behavior. In conclusion, the overall risks/benefits tradeoff is key in predicting vaccination behavior and intention, but the concerns for the baby weigh more than those for the mother in the decision, shedding light on this previously neglected aspect.

## 1. Introduction

While an individual’s vaccination involves a trade-off between risks and benefits for the single individual, pregnant women have to consider the benefits and risks not only for themselves, but also for the baby they are carrying. Additionally, breastfeeding women have to consider the benefits and risks for themselves and their nursling. Comparing the two groups may help reveal the similarities or differences of the decision process; indeed, the risks for a nursling are usually smaller than those for the fetus, and, also, the potential risks for the mother do not directly affect the baby [1].

The psychological literature suggests that risk perception plays an important role in preventive behaviors, including immunization [2], and events or stimuli are often judged based on the positive–negative feelings they evoke (affect heuristic [3]). Indeed, many studies preceding the COVID-19 pandemic showed that being concerned about the risks for the baby is the most common barrier to vaccination during pregnancy [4,5,6], but, also, vice versa, the desire to protect the baby is the most common facilitator [4,7,8]. When compared directly, the concern for the safety of the vaccine for the baby is cited as a primary concern more frequently (95%) than the safety for the mother (82%) [9]. A systematic review of 120 articles and a meta-analysis of 49 of them [10] showed that both the benefits for the baby and those for the mother were similarly predictive of vaccine uptake, whereas the concern for the risks for the baby (referred to as “risk of vaccine harm during pregnancy”) had a stronger negative effect on vaccine uptake than the concern for the risks of side effects for the mother [10]. However, it remains unclear how the interplay between perceived benefits and risks for the mother and baby contributes to the decision to receive a vaccination during pregnancy or lactation.

Several studies have examined the acceptability of COVID-19 vaccines in pregnant and (to a lesser extent) in breastfeeding women, showing a high heterogeneity in vaccine acceptance and hesitancy in several countries and at different timing of the pandemic [11,12,13]. At the same time, most studies showed that the main reasons for vaccine hesitancy in pregnant and breastfeeding women were similar to those expressed by the general population. Specifically, they included concerns over safety and fear of adverse events and lack of information or lack of recommendation from healthcare professionals [11,12,13,14]. These reasons were amplified by the lack of safety data for pregnant women and also by concerns about the possibility of harm to the fetus [15,16] and long-term adverse events in children, including children of breastfeeding women [17]. Conversely, factors favoring the acceptance of the COVID-19 vaccination were: trust in the importance (i.e., knowing the risks of the illness) and effectiveness of the COVID-19 vaccine and other vaccines and, more generally, trust in health institutions [13]. However, no study has examined in details the role of the perceived risks and benefits for the mother and for the baby.

The aim of the present study was to examine the role of the perception of risks and benefits for the mother and her babies in deciding about the COVID-19 vaccination in pregnant and breastfeeding women. Based on the literature, it was expected that:

**H1:**  *The mother’s status is predictive of COVID-19 vaccination self-reported behavior and intention: pregnant women are less likely than breastfeeding women to have been vaccinated and to intend to be vaccinated while in the current status.*

Indeed, in earlier studies, vaccine hesitancy was higher in pregnant women than in breastfeeding women [18,19], although later studies showed more mixed results and a high heterogeneity [12]. Earlier on, pregnant women were less likely to accept a vaccine (52% vs. 73%), they were more often concerned about possible harmful side effects for their baby (66% vs. 28%), and they were more interested in safety and effectiveness data specific for them (49% vs. 33%) than were mothers considering vaccinating their children [19]. Similarly, in a large study in six European countries, pregnant women were less likely to accept a vaccine than breastfeeding women (62% vs. 69%) [18].

**H2:**  *Similarly to the general population, COVID-19 risk perception is predictive of COVID-19 vaccination self-reported behavior and intention: women who (a) are more worried about COVID-19 (b) perceive themselves as more likely to become infected, (c) perceived it as a severe disease, and (d) are more concerned about variants, and they are more likely to be vaccinated or intending to become vaccinated.*

Indeed, COVID-19 risk perception has been repeatedly found to be a predictor of vaccine acceptance in the general population [20,21,22] and also in pregnant women some evidence suggests this link [13] although this seems more linked to an emotional level than a cognitive level of risk perception as only a very weak positive correlation was found between C19 knowledge and C19 vaccine acceptance [12].

**H3:**  *The trade-off between the perceived risks and the perceived benefits of vaccination is predictive of COVID-19 vaccination self-reported behavior and intention, regardless of the specific consideration for mother and baby. When the benefits of vaccination clearly outweigh the risks, women are more likely to be vaccinated or intend to be vaccinated than when the risks outweigh the benefits or they are similar.*

**H4:**  *The concerns for the baby are more important than those for the mother in predicting vaccination behavior and intention. For the same value of trade-off between risks and benefits of vaccination, as the risks for the baby increase, the likelihood that women have been vaccinated or intend to be vaccinated decreases.*

Both these predictions are based on the literature reviewed above about the role of perceived benefits and risks, especially on the review and meta-analysis by Kilich and colleagues [10].

**H5:**  *During pregnancy, the concerns for the baby weigh more than during breastfeeding in the decision to become vaccinated.*

This prediction stems from H1 and from considerations about the actual potential for risks of adverse events for the fetus and the nursling.

## 2. Materials and Methods

### 2.1. Participants

Participants were recruited through social media posts, with a link to an online questionnaire, and informed consent was obtained from all women involved in the study. Data were collected nation-wide between late July and early September 2021. Inclusion criteria were being pregnant, breastfeeding, or both. The study information sheet was read by 1720 potential participants, and 1484 consented to participate. Of those, 52 (3.5%) participants did not meet the inclusion criteria, 270 (18.2%) participants dropped out during the survey, 50 (3.4%) were excluded because they were vaccinated before being pregnant or before knowing they were pregnant, and eight (0.5%) were excluded because they provided incoherent answers, leaving 1104 (77%) participants for the analyses. The final sample consisted of 572 (52%) breastfeeding women and 532 (48%) pregnant women (of whom 34 were both pregnant and breastfeeding). The study was approved by the ethical committee for psychological studies of the first authors’ university (protocol: 4220, approved 7 July 2021).

### 2.2. Procedure

Participants who consented to participate indicated whether they were pregnant and whether they were breastfeeding. They were then asked personal information, including: age, level of education (middle school, high school, university degree, higher degrees), employment (employee, unemployed, freelancer), and whether they had other children (no, one, two, or more). Pregnant women were asked to report their current pregnancy week. Breastfeeding women were asked the age of the breastfed baby. The *C19 Vaccine Status* was assessed by asking all women if they had received at least one dose of a COVID-19 vaccine (Yes, No) during pregnancy and/or during breastfeeding. Women who had not yet been vaccinated were asked to indicate their *willingness to become vaccinated (WTV)*, i.e., how likely they were to become vaccinated against COVID-19 (from 0 = *Not at all likely* to 100 = *Extremely likely*), with a vaccine recommended for their case. This question was asked twice, once referring to their status at the time of the questionnaire and once referring to their intention if they were not pregnant nor breastfeeding. Four questions assessed the perception of benefits and risks for the mother and for the baby associated with the mother’s COVID-19 vaccination (all measured on a scale from 1 = *Completely disagree* to 5 = *Completely agree*). These four questions were combined in two indexes (see also Figure S1):

(1) $$C19\;Vaccination\;risks/benefits\;overall\;ratio=\frac{\left(risk\;for\;baby+risk\;for\;mother\right)}{\left(benefit\;for\;baby+benefit\;for\;mother\right)}$$

The index *C19 Vaccination risks/benefits overall ratio* (1) can range from a minimum of 0.2 (when risks are both judged to be equal to the minimum value of 1 and benefits are both judged to be equal to the maximum value of 5) to a maximum of 5 (when risks are both equal to 5 and benefits are both equal to 1). The index is equal to 1 when risks and benefits are judged to be equal overall; values smaller than 1 indicate lower risks than the benefits, whereas values bigger than 1 indicate that the risks are judged higher than the benefits.

(2) $$C19\;Vaccination\;baby/mother\;risk/benefit\;ratio=\frac{\frac{risk\;for\;baby}{benefit\;for\;baby}}{\frac{risk\;for\;mother}{benefit\;for\;mother}}$$

The index *C19 Vaccination baby/mother risk/benefit ratio* (2) can range from a minimum of 0.04 (when the risk/benefit ratio for the baby is equal to the minimum of 0.2 and the risk/benefit ratio for mother is equal to the maximum of 5), to a maximum of 25 (when risk/benefit ratio for the baby has the highest possible value of 5 and that for the mother the lowest possible value of 0.2). The index is equal to 1 when the two risks-benefits ratios are judged to be equal; values lower than 1 indicate a lower risks-benefits ratio for the baby than for the mother (e.g., when the benefits are perceived as equal, the risks for the mother are higher than those for the baby), and values higher than 1 indicate the opposite (risks–benefits ratio higher for the baby than for the mother).

Similarly to previous studies [20,23], COVID-19 risk perception was assessed by asking participants to report their perceived severity of the disease, the perceived likelihood of being infected, how scared they felt about the disease, and the concern for possible variants (all measured on a scale from 0 = *Not at all* to 100 = *Extremely*). Participants were then asked to complete the Pandemic Fatigue scale, assessing a general distress and sense of fatigue related to the pandemic, indicating their degree of agreement (1 = *Not at all*; 7 = *Very much*) with six items, such as “I feel challenged by following all of the rules and behavioral rules regarding C19.” [24]. Further, participants completed a previously used ad hoc scale, investigating the sense of conspiracy related to the COVID-19 context, indicating their degree of agreement (1 = *Not at all*; 7 = *Very much*) with seven items, such as “The C19 virus was created in a laboratory” and “Vaccines against C19 can alter people’s DNA” [25]. Finally, participants were asked to answer an eight-item scale to assess the perception of vaccines in general, indicating their agreement (1 = *Not at all*; to 5 = *Very much*) on items such as “Vaccines are important to human health” and “Vaccines are produced and recommended only for the economic interest of pharmaceutical companies” [26].

### 2.3. Statistical Analyses

#### 2.3.1. Descriptive Analysis

The variables in the study were summarized by frequency, for categorical variables, as well as median (and Inter Quartile Range, IQR) for continuous variables (see Table 1). Wilcoxon rank sum tests were computed to compare variables on an ordinal Likert scale or on continuous scores across mother status (pregnancy or breastfeeding), while, for categorical variables, the Pearson chi-squared test was used. Statistical significance was assumed at the 5% level. Tables S1–S4 provide additional descriptive analyses not reported in the main article.

#### 2.3.2. Dimensionality Reduction—Factor Analyses

Four different factor analyses were performed for the Pandemic Fatigue scale [24], as well as for the group of variables related to COVID-19 risk perception [20,23], COVID-19 conspiracy [25], and to vaccine perception in general [26]. For all the factor analyses, the amount of variance explained by the one factor solution was acceptable (see Table S5).

#### 2.3.3. Logistic and Beta Regressions

To estimate the influence of the perceived risks/benefits ratios (overall and baby/mother) from COVID-19 vaccination on the probability to have received the vaccine against COVID-19, a logistic regression model was employed, in which the dependent variable was the COVID-19 Vaccine Status (0 = not yet received; 1 = received). Covariates (mother status, vaccine perception, COVID-19 risk perception, COVID-19 conspiracy, and pandemic fatigue) were included, minimizing the AIC index with a forward selection criteria. The presence of interactions between covariates and risks/benefits ratio indices was tested employing a Chi-squared test, fixing a significance level equal to 5%. The model included the mother’s age (in continuous form), the educational level, the employment, and the presence of other kids to adjust for non-probability sampling. The results are presented by means of Odds Ratios (ORs) by exponentiating the estimated coefficients from the logistic regression, calculating the relative 95% Confidence Interval (95%CI).

To evaluate which factors influenced the WTV among mothers who had not yet received the vaccine, a beta regression model, which is commonly used to model variables that assume values in the standard unit interval (0,1), was used. The WTV was divided by 100, applying to the new scale correction [27] to have values strictly between 0 and 1, extremes excluded. Two separate models were estimated, one for the WTV in the current status and one for the WTV assuming not to be pregnant and/or in breastfeeding. For these models, the same selection variables scheme adopted for the logistic regression was considered. The results are presented using ORs by exponentiating the estimated coefficients reporting the relative 95%CI.

Regression analyses were performed by R 4.2 statistical software using the package betareg for the beta regression model.

## 3. Results

### 3.1. Demographic

The main characteristics of the sample are reported in Table 1.

Mothers reported an average age of 34.2 years (min–max: 20–48 years), with a predominant high educational level (53% and 16% obtained a degree or a higher education, respectively). The majority was employed as a private or public employee (67%) and had no other child (44%) or one other child (43%). With respect to their mother status, pregnant women were between six and forty-three weeks of gestation (average: 28.4, Standard Deviation (SD): 9.21), while, for breastfeeding women, 25% of the lactated children were below three months, 25% were between four and eight months, 25% were between nine and fifteen months, and 5.3% were older than three years.

**Figure 1:**
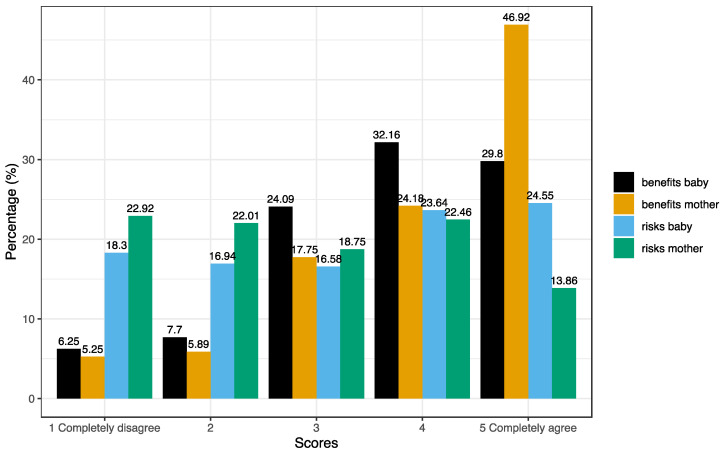
Distribution of the scores of the perception of COVID-19 vaccination risks and benefits for the baby and the mother, each assessed on a scale from 1 (=completely disagree) to 5 (=completely agree).

The distribution of the scores for the perceived risks and benefits for the baby and the mother is depicted in Figure 1. All scores differed between vaccinated and unvaccinated women (Table 2). Both overall and baby/mother risks/benefits ratios differed between vaccinated and unvaccinated women and between pregnant and breastfeeding women (Table 3).

### 3.2. Logistic Regression Model

The factors associated with a reduction in the probability to be vaccinated (Table 4) were: the current status, with pregnant women reporting a heavy reduction (−73%) in the probability to be vaccinated relative to those breastfeeding (OR: 0.27, 95%CI: 0.10–0.76), perceiving that, overall, the vaccination risks exceed the benefits (+1 point increase in the risks/benefits overall index: OR: 0.19, 95%CI: 0.04–0.74), and having a higher COVID-19 conspiracy score (+1 point increase: OR: 0.36, 95%CI: 0.24–0.54). Whereas higher values on the pandemic fatigue scale were associated with an increased probability to become vaccinated (+1 point increase: OR: 1.44, 95%CI: 1.13–1.83).

**Figure 2:**
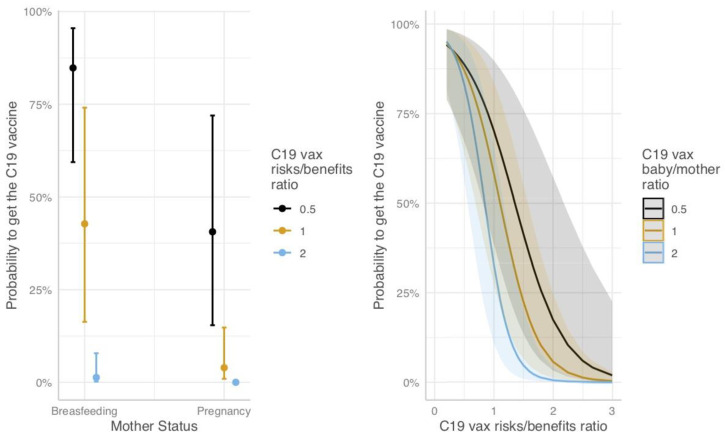
Predicted probability and 95%CI to be vaccinated, considering the interaction between mother status and COVID-19 vaccination risks/benefits ratio (**left**) and COVID-19 vaccination risks/benefits ratio and COVID-19 vax baby/mother ratio (**right**).

Although no marginal effect was found for the COVID-19 vaccination baby/mother risks/benefits ratio, results showed a significant interaction between this index and COVID-19 vaccination risks/benefits overall ratio, leading mothers who perceived a higher risks/benefits ratio for their baby than for themselves to be more hesitant (OR: 0.23, 95%CI: 0.08–0.60; Figure 2, left panel). Further, COVID-19 vaccination risks/benefits overall ratio also interacted with the mother status, accentuating the reduction in the likelihood of being vaccinated among pregnant women (OR: 0.20, 95%CI: 0.05–0.82; Figure 2, right panel).

### 3.3. Beta Regression Models

As shown in Table 5, the higher the risks/benefits overall ratio, the lower the WTV of unvaccinated women, both at the time of the survey (+1 point increase: OR: 0.83, 95%CI: 0.73–0.94) and if they were not pregnant or breastfeeding (+1 point increase: OR: 0.79, 95%CI: 0.69–0.90). The risks/benefits baby/mother ratio decreased the WTV in the current status (+1 point increase: OR: 0.87, 95%CI: 0.83–0.92), while it slightly increased if women were neither pregnant nor breastfeeding (+1 point increase: OR: 1.05, 95%CI: 1.00–1.11). Being pregnant decreased the WTV in the current status (+1 point increase: OR: 0.61, 95%CI: 0.49–0.76), whereas having a general attitude towards vaccination increased the WTV if not pregnant or breastfeeding (+1 point increase: OR: 1.18, 95%CI: 1.02–1.36). C19 Risk perception increased WTV in both models, respectively (+1 point increase: OR: 1.24, 95%CI: 1.20–1.39 for current status and OR: 1.43, 95%CI: 1.27–1.61 if not pregnant or breastfeeding); to the contrary, COVID-19 conspiracy decreased the WTV in both models, respectively (OR: 0.57, 95%CI: 0.49–0.67 for current status and OR: 0.59, 95%CI: 0.49–0.71 if not pregnant or breastfeeding).

## 4. Discussion

In a convenience sample of over a thousand women, this study examined the interplay of perceived risks and benefits for the mother and the baby in deciding to become vaccinated against C19 while pregnant or breastfeeding, confirming most of the hypotheses based on the literature and highlighting some important relationships.

In the present sample, only about a quarter of pregnant women had received the vaccine while pregnant, whereas about two thirds of breastfeeding women received it while breastfeeding. In other words, pregnant women were less likely to be vaccinated or to be willing to become vaccinated than breastfeeding women (supporting H1, in line with [18,19]), but they were equally likely to intend to be vaccinated if they were not pregnant, suggesting that their attitude is attributable to their current status. This result is further supported by the finding that a general positive attitude towards immunization was a positive predictor only when modeling the intention to become vaccinated if not pregnant or breastfeeding. These findings suggest that the hesitancy shown by pregnant women in our sample was highly context-specific and temporary. It remains to be ascertained whether this type of hesitancy could still affect attitudes towards other vaccines and childhood vaccines, which are often formed during pregnancy [28].

In line with the literature [2,20,21,22], findings also confirmed that COVID-19 risk perception plays a role in women’s WTV, with higher risk perception yielding a higher intention to become vaccinated, both in the current status and if not pregnant or breastfeeding. However, COVID-19 risk perception was not a predictor of actually being vaccinated at the time of the survey, only partially supporting H2. A possible explanation is that, after being vaccinated, women’s COVID-19 risk perception decreased, showing no predictive value. In other words, if COVID-19 risk perception was measured before these women were vaccinated, it is expected that it would predict the decision to become vaccinated. This interpretation is supported by evidence showing that COVID-19 risk perception decreases after being vaccinated [29].

The predictive power of the trade-off between perceived risks and benefits was confirmed in all three models, fully supporting H3. The higher the risks–benefits trade-off, the lower the probability that women have been vaccinated against C19 while pregnant or breastfeeding and, if not yet vaccinated, the lower their willingness to be vaccinated, both at the time of the survey and if they were not pregnant or breastfeeding. This is in line with the psychological literature on the affect heuristic [3], whereby people often heavily rely on their feelings to make judgments and decisions, in this case on the perceived risks and benefits of the vaccination.

The distinctive role of the risks–benefit ratio for the baby and for the mother also emerged in the analyses, supporting H4. This finding is corroborated by the interaction found in the logistic model: the higher the baby–mother ratio, the steeper the drop in the probability of being vaccinated when the risks–benefits ratio increases (Figure 2, right panel). For example, for women whose general risks–benefits trade-off is equal to 1, the probability of being vaccinated against C19 is around 55–60% when the baby–mother ratio is also equal to 1. It lowers to around 35% when the baby–mother ratio is equal to 2 (i.e., when the risks–benefits trade-off for the baby is twice the risks–benefits trade-off for the mother). Whereas it reaches about 70% when the baby–mother ratio is equal to 0.5 (i.e., when the risks–benefits trade-off for the mother is twice the risks–benefits trade-off for the baby). This finding suggests that, all other things being equal, an increase in the perception of risks for the baby weighed more against the decision to vaccinate than a similar increase in the perception of risks for the mother, shedding light on this previously neglected aspect [10].

Among unvaccinated women, the risks–benefits ratio for the baby and for the mother had a direct effect on the intention to become vaccinated at the time of the survey: the higher the baby–mother ratio, the lower the intention to become vaccinated while being pregnant or breastfeeding (in line with H4). However, the opposite was found when modeling the intention to become vaccinated if women were not pregnant or breastfeeding. Considering that most unvaccinated women were pregnant, this result suggests that the relevance of the risks for the baby in the decision decreases once a woman is no longer pregnant, providing support for H5. Additionally, an interaction between the mother’s current status and the risks–benefits trade-off was found: the level of risks–benefits ratio being equal, breastfeeding women are more likely to be vaccinated than pregnant women, and the effect of the risks–benefits ratio is stronger in breastfeeding women (Figure 2, left panel). For example, when the benefits of the vaccination are judged to be two times greater than the risks, on average, lactating women are around 85% likely to have been vaccinated, whereas pregnant women are around 40% likely to have been vaccinated. When the benefits are judged to be equal to the risks, the probability of breastfeeding women to be vaccinated is around 40–45%, but, for pregnant women, it is around 5%. While not directly expected, this is in line with H5, as, for a pregnant woman, the benefits have to outweigh the risks more than for a breastfeeding woman to have decided to vaccinate.

Moreover, COVID-19 conspiracy was a consistent predictor in all models: the higher the conspiracy score, the lower the probability that women were vaccinated and their WTV now or if not pregnant/breastfeeding. This is not surprising, as conspiracy mentality has been repeatedly associated with vaccine hesitancy in general [30,31] in the COVID-19 context [32,33] and also during pregnancy [34].

Finally, the level of pandemic fatigue (i.e., being tired of information about COVID-19 and of behavioral measures to counter it [24]) was positively associated with having been vaccinated during pregnancy or breastfeeding, but not with WTV among unvaccinated women. This seems to contrast with previous findings, showing that pandemic fatigue is a strong predictor of non-adherence to health protective measures [24]. However, it is possible that high pandemic fatigue leads to avoidance of information and to a reluctance to adhere to behavioral measures, such as wearing masks, keeping distance, and washing hands, but vaccination could be seen as a solution to the pandemic [35]; this would explain why a positive association between pandemic fatigue and vaccine uptake was found.

This study has limitations that should be considered. While the sample recruited was not representative of the population of pregnant and lactating women, the sample reached was ample and varied, and the use of social media through which participants were recruited has become even more widespread than in the past. Nonetheless, also due to the drop-out of about 25% of participants during the questionnaire, the generalizability of the findings should be taken cautiously and compared with other findings. However, in the regression analyses, all the confounders (age, educational level, employment, and presence of other children) were included to adjust the effect estimate. Finally, as the pandemic is constantly evolving, and the recommendations for immunization against C19 during pregnancy have changed since the study was conducted (when it was still recommended to discuss with healthcare professionals the benefits and risks of C19 vaccination for each pregnant or breastfeeding woman), it is important to consider the context when data were collected when interpreting the findings.

## 5. Conclusions

To summarize and conclude, the study focused on pregnant and breastfeeding women’s decision making about COVID-19 vaccination, especially about the role of the perception of risks and benefits for themselves and for their babies. The COVID-19 vaccination risks/benefits tradeoff was highly predictive of behavior and intention. Ceteris paribus, an increase in the perception of risks for the baby weighed more against the decision to vaccinate than a similar increase in the perception of risks for the mother, shedding light on this previously neglected aspect. When counseling pregnant and breastfeeding women about vaccinations, it is important to be aware that their decision is likely based on the risks and benefits tradeoff in general, but also that they are particularly worried about the baby and may not fully appreciate the indirect benefits to the baby conveyed by the mother’s vaccination. Indeed, it is also possible that women underappreciate the benefits for their babies in vaccinating themselves, as being less likely to experience severe COVID-19 illness also reduces the risks of negative events for the fetus (for pregnant women) and makes them more available to care for their babies (for breastfeeding women). While pregnant women’s hesitancy seems transient, it is important to foster decision-making, improving women’s understanding and awareness of the risks and benefits for them and for their babies and helping women to weighing them.

## Figures and Tables

**Table 1:** Main characteristics of the mothers by COVID-19 vaccine status.

Variable	Overall, N = 1104 ^1^	C19 Vaccine Status		*p*-Value ^2^
		Not Vaccinated, N = 592 ^1^	Vaccinated, N = 512 ^1^	
**Age** (years)	34.0 (31.0, 37.0)	34.0 (31.0, 37.0)	35.0 (32.0, 37.0)	**0.012**
**Education**				**<0.001**
Middle school	25 (2.3%)	15 (2.5%)	10 (2.0%)	
High school	318 (29%)	212 (36%)	106 (21%)	
University degree	581 (53%)	289 (49%)	292 (57%)	
Higher level degree	180 (16%)	76 (13%)	104 (20%)	
**Employment**				0.516
Private or public employee	735 (67%)	392 (66%)	343 (67%)	
Unemployed or Other	182 (16%)	104 (18%)	78 (15%)	
Self-employed	187 (17%)	96 (16%)	91 (18%)	
**Other Children**				0.238
No	476 (43%)	266 (45%)	210 (41%)	
1	481 (44%)	244 (41%)	237 (46%)	
2+	147 (13%)	82 (14%)	65 (13%)	
**Mother Status**				**<0.001**
Breastfeeding	572 (52%)	188 (32%)	384 (75%)	
Pregnacy	532 (48%)	404 (68%)	128 (25%)	
**C19 Risk Perception**	0.19 (−0.58, 0.70)	−0.19 (−1.09, 0.58)	0.41 (−0.02, 0.77)	**<0.001**
**Pandemic Fatigue**	0.04 (−0.62, 0.64)	0.21 (−0.50 0.79)	−0.18 (−0.68, 0.39)	**<0.001**
**Pro-vax Attitude**	0.17 (−0.57, 0.74)	−0.25 (−0.96, 0.47)	0.48 (0.01, 0.99)	**<0.001**
**C19 Conspiracy score**	−0.30 (−0.79, 0.66)	0.51 (−0.36, 1.26)	−0.70 (−0.96, −0.32)	**<0.001**

^1^ Median (IQR) or frequency (%). ^2^ Wilcoxon rank sum test; Pearson’s chi-squared test. Abbreviation: C19 for COVID-19, vax for vaccination. Bold: statistically significant results.

**Table 2:** COVID-19 vaccine perception of risks and benefits for the baby and the mother by COVID-19 vaccine status.

C19 Vaccine Perception of	Overall, N = 1104 ^1^	C19 Vaccine Status		*p*-Value ^2^
		Not Vaccinated, N = 592	Vaccinated, N = 512 ^1^	
Risks for baby	3 (2, 4)	4 (4, 5)	2 (1, 3)	**<0.001**
Risks for mother	3 (2, 4)	4 (2, 4)	2 (1, 3)	**<0.001**
Benefits for baby	4 (3, 5)	3 (3, 4)	5 (4, 5)	**<0.001**
Benefits for mother	4 (3, 5)	4 (3, 4)	5 (5, 5)	**<0.001**

^1^ Median (IQR) or frequency (%). ^2^ Wilcoxon rank sum test. Abbreviation: C19 for COVID-19. Bold: statistically significant results.

**Table 3:** C19 Vaccination risks/benefits ratio and COVID-19 vaccination baby/mother ratio by mother status and COVID-19 vaccine status.

C19 Vax	Mother Status				C19 Vaccine Status		
	Overall, N = 1104 ^1^	Breasfeeding, N = 572 ^1^	Pregnancy, N = 532 ^1^	*p*-Value ^2^	Not Vaccinated, N = 592 ^1^	Vaccinated, N = 512 ^1^	*p*-Value ^2^
Risks/benefits ratio	0.75 (0.44, 1.17)	0.62 (0.40, 1.00)	0.89 (0.56, 1.33)	<0.001	1.14 (0.80, 1.60)	0.44 (0.30, 0.67)	<0.001
Baby/mother ratio	1.00 (1.00, 2.00)	1.00 (0.83, 1.67)	1.25 (1.00, 2.00)	<0.001	1.25 (1.00, 2.02)	1.00 (0.83, 1.33)	<0.001

^1^ Median (IQR) or frequency (%). ^2^ Wilcoxon rank sum test. Abbreviation: C19 for COVID-19; vax for vaccination. Bold: statistically significant results.

**Table 4:** Odds ratios (ORs) estimated by a logistic regression model for the probability to be vaccinated by the COVID-19 vaccine with respect to the reference category ^1^.

Predictors	OR	95%CI	*p*-Values
COVID-19 vax risks/benefits overall ratio	0.19	0.04–0.74	**0.021**
COVID-19 vax baby/mother risks/benefits ratio	1.52	0.81–2.90	0.193
Mother Status [Pregnancy]	0.27	0.10–0.76	**0.012**
Age (+1 years)	1.04	0.98–1.09	0.175
Education [High school]	0.89	0.23–3.15	0.858
Education [University Degree]	1.04	0.27–3.63	0.955
Education [High level degree]	1.10	0.27–4.21	0.891
Employment [Unemployed or Other]	1.14	0.64–2.03	0.666
Employment [Self-employed]	1.21	0.69–2.14	0.512
Other Children [1]	1.03	0.66–1.59	0.905
Other Children [2+]	0.81	0.42–1.55	0.513
COVID-19 Conspiracy score ^2^	0.36	0.24–0.54	**<0.001**
Pandemic Fatigue ^2^	1.44	1.13–1.83	**0.003**
COVID-19 vax risks/benefits ratio^×^Mother Status [Pregnancy]	0.20	0.05–0.82	**0.029**
COVID-19 vax risks/benefits ratio^×^COVID-19 vax baby/mother ratio	0.23	0.08–0.60	**0.003**
Observations	1104		
R^2^ Tjur	0.636		

^1^ Mother Status [Breastfeeding], Education [Middle School], Employment [Private or public employee], Other Children [No]. ^2^ 1-point increase. Abbreviation: C19 for COVID-19; vax for vaccination. Bold: statistically significant results.

**Table 5:** Odds Ratio, estimated by a beta regression model for the willingness to become vaccinated in the current status and if not pregnant/breastfeeding, with respect to the reference category ^1^.

Predictors	WTV in Current Status			WTV if Not Preg./Breast.		
	OR	95%CI	*p*-Values	OR	95%CI	*p*-Values
COVID-19 vax risks/benefits ratio	0.83	0.73–0.94	**0.003**	0.79	0.69–0.90	**<0.001**
COVID-19 vax baby/mother ratio	0.87	0.83–0.92	**<0.001**	1.05	1.00–1.11	**0.045**
Mother Status [Pregnancy]	0.61	0.49–0.76	**<0.001**	NS	-	-
Age (+1 years)	0.99	0.96–1.01	0.234	0.98	0.95–1.00	0.075
Education [High school]	0.68	0.36–1.30	0.246	0.97	0.51–1.84	0.924
Education [University Degree]	0.74	0.39–1.41	0.362	0.86	0.45–1.64	0.643
Education [High level degree]	0.78	0.39–1.57	0.487	0.96	0.48–1.91	0.898
Employment [Unemployed or Other]	0.98	0.74–1.29	0.882	0.90	0.69–1.18	0.460
Employment [Self-employed]	0.84	0.63–1.11	0.223	0.89	0.67–1.17	0.401
Other Children [1]	1.03	0.82–1.29	0.804	0.94	0.75–1.16	0.548
Other Children [2+]	1.11	0.81–1.53	0.517	0.83	0.61–1.14	0.247
COVID-19 Risk Perception ^2^	1.24	1.10–1.39	**<0.001**	1.43	1.27–1.61	**<0.001**
COVID-19 Conspiracy score ^2^	0.57	0.49–0.67	**<0.001**	0.59	0.49–0.71	**<0.001**
Pro-vax Attitude ^2^	NS	-	-	1.18	1.02–1.36	**0.026**
Observations	592			592		
R^2^	0.406			0.611		

^1^ Mother Status [Breastfeeding], Education [Middle School], Employment [Private or public employee], Other Children [No]. ^2^ 1-point increase. Abbreviation: C19 for COVID-19; vax for vaccination. Bold: statistically significant results.

## Data Availability

Data are available upon request to the authors.

## Supplementary Materials

The following supporting information can be downloaded at: https://www.mdpi.com/article/10.3390/jcm12103469/s1, Table S1. Selection Criteria. Questionnaire completion of at least 85%, vaccination before pregnancy, and completeness of the analyzed variables; Table S2. Main characteristics by Mother Status; Table S3. Main characteristics of not vaccinated mothers by Mother Status; Table S4. C19 vaccine perception of Risks and Benefits for baby and mother by Mother Status; Table S5. Dimensionality reduction—factor analyses; Figure S1. Joint and marginal distribution of the scores for C19 vax risks/benefits ratio and C19 vax baby/mother ratio. With the dotted lines the ratios equal to the unity.
