# Supplementary material for: The Interplay of Perceived Risks and Benefits in Deciding to Become Vaccinated against COVID-19 While Pregnant or Breastfeeding: A Cross-Sectional Study in Italy
Source: J Clin Med. 2023 May 15;12(10):3469. doi: 10.3390/jcm12103469 (PMC10219324; doi:10.3390/jcm12103469)
Supplement: Supplementary file 1 [file jcm-12-03469-s001.zip › jcm-2291336-supplementary.pdf]

Supplementary Material

The Interplay of Perceived Risks and Benefits in Deciding to Become Vaccinated against COVID-19 While Pregnant or Breastfeeding: A Cross-Sectional Study in Italy

Tables

Table S1. Selection Criteria. Questionnaire completion of at least 85%, vaccination before pregnancy, and completeness of the analyzed variables.

**Variable, n (%)**	**Overall**, N = 1,432	**C19 Vaccine Status**		
		Not vaccinated, N = 725[1]	Vaccinated, N = 638[1]	Missing, N = 69[1]
Completion <85%	251 (18%)	112 (15%)	70 (11%)	69 (100%)
Vaccinated before pregnancy	65 (4.5%)	0 (0%)	65 (10%)	0 (0%)
Missing values	278 (19%)	133 (18%)	76 (12%)	69 (100%)
Questionnaires included	1,104 (77%)	592 (82%)	512 (80%)	0 (0%)

Table S2. Main characteristics by Mother Status.

Variable	Overall, N = 1,104[1]	Mother Status		*P*-value[2]
		**Breastfeeding**, N = 572[1]	**Pregnancy**, N = 532[1]	
**Age** (years)	34.0 (31.0, 37.0)	35.0 (32.0, 38.0)	34.0 (31.0, 37.0)	<0.001
**Education**				<0.001
Middle school	25 (2.3%)	10 (1.7%)	15 (2.8%)	
High school	318 (29%)	136 (24%)	182 (34%)	
University degree	581 (53%)	319 (56%)	262 (49%)	
Higher level degree	180 (16%)	107 (19%)	73 (14%)	
**Employment**				0.167
Private or public employee	735 (67%)	366 (64%)	369 (69%)	
Unemployed or Other	182 (16%)	102 (18%)	80 (15%)	
Self-employed	187 (17%)	104 (18%)	83 (16%)	
**Other Children**				<0.001
No	476 (43%)	221 (39%)	255 (48%)	
1	481 (44%)	255 (45%)	226 (42%)	
2+	147 (13%)	96 (17%)	51 (9.6%)	
**C19 Risk Perception**	0.19 (-0.58, 0.70)	0.23 (-0.45, 0.72)	0.13 (-0.71, 0.70)	0.065
**Pandemic Fatigue**	0.04 (-0.62, 0.64)	0.07 (-0.57, 0.62)	-0.02 (-0.64, 0.69)	0.536

**Pro-vax Attitude**	0.17 (-0.75, 0.74)	0.21 (-0.45, 0.74)	0.07 (-0.68, 0.72)	0.061
**C19 Conspiracy**	-0.30 (-0.79, 0.66)	-0.40 (-0.84, 0.38)	-0.09 (-0.72, 0.80)	<0.001

[1] Median (IQR) or Frequency (%)

[2] Wilcoxon rank sum test; Pearson's Chi-squared test

Table S3. Main characteristics of not vaccinated mothers by Mother Status.

Variable	Overall, N = 592[1]	Mother Status		*P*-value[2]
		Breastfeeding, N = 188[1]	Pregnancy, N = 404[1]	
**WTV in current status**	10 (0, 51)	20 (0, 70)	10 (0, 50)	0.117
**WTV if not breastfeeding/pregnant**	94 (30, 100)	73 (10, 100)	100 (50, 100)	<0.001
**Age** (years)	34.0 (31.0, 37.0)	35.0 (31.8, 38.0)	34.0 (30.0, 37.0)	0.010
**Education**				0.333
Middle school	15 (2.5%)	4 (2.1%)	11 (2.7%)	
High school	212 (36%)	59 (31%)	153 (38%)	
University degree	289 (49%)	96 (51%)	193 (48%)	
Higher level degree	76 (13%)	29 (15%)	47 (12%)	
**Employment**				0.038
Private or public employee	392 (66%)	112 (60%)	280 (69%)	
Unemployed or Other	104 (18%)	43 (23%)	61 (15%)	
Self-employed	96 (16%)	33 (18%)	63 (16%)	
**Other Children**				<0.001
No	266 (45%)	67 (36%)	199 (49%)	
1	244 (41%)	80 (43%)	164 (41%)	
2+	82 (14%)	41 (22%)	41 (10%)	
**C19 Risk Perception**	-0.19 (-1.09, 0.58)	-0.45 (-1.34, 0.35)	-0.09 (-0.82, 0.65)	0.002
**Pandemic Fatigue**	0.21 (-	0.43 (-0.23,	0.11 (-0.62,	0.004

	0.50, 0.79)	0.93)	0.74)	
**Pro-vax Attitude**	-0.25 (-0.96, 0.47)	-0.48 (-1.24, 0.20)	-0.16 (-0.87, 0.54)	<0.001
**C19 Conspiracy**	0.51 (-0.36, 1.26)	0.86 (0.06, 1.40)	0.33 (-0.46, 1.16)	<0.001

[1] Median (IQR) or Frequency (%)

[2] Wilcoxon rank sum test; Pearson's Chi-squared test

Table S4. C19 vaccine perception of Risks and Benefits for baby and mother by Mother Status.

		Mother Status		P-value[2]
**C19 vaccine perception of**	**Overall**, N = 1,104[1]	Breastfeeding, N = 572	Pregnancy, N = 532[1]	
Risks for baby	3 (2, 4)	3 (2, 4)	4 (2, 5)	<0.001
Risks for mother	3 (2, 4)	3 (2, 4)	3 (2, 4)	0.020
Benefits for baby	4 (3, 5)	4 (3, 5)	4 (3, 4.25)	<0.001
Benefits for mother	4 (3, 5)	5 (4, 5)	4 (3, 5)	<0.001

[1] Median (IQR) or Frequency (%)

[2] Wilcoxon rank sum test

Table S5. Dimensionality reduction - factor analyses.

	COVID-19 perceived risk	Pandemic fatigue scale	Vaccine perception	COVID-19 conspiracy
**Num. of items**	4	6	8	7
**Cronbach's α**	0.85	0.79	0.88	0.85
**Item 1**	Scariness	Pfs 1	Vax perception 1	C-19 Conspiracy 1
**Item 2**	Severity	Pfs 2	Vax perception 2	C-19 Conspiracy 2
**Item 3**	Contagiousness	Pfs 3	Vax perception 3	C-19 Conspiracy 3
**Item 4**	Mutation	Pfs 4	Vax perception 4	C-19 Conspiracy 4
**Item 5**		Pfs 5	Vax perception 5	C-19 Conspiracy 5_rev
**Item6**		Pfs 6	Vax perception 6	C-19 Conspiracy 6_rev
**Item7**			Vax perception 7	C-19 Conspiracy 7_rev
**Item 8**			Vax	

			perception 8	
**Load. item 1**	0.89	0.64	0.70	0.96
**Load. item 2**	0.83	0.79	0.75	0.46
**Load. item 3**	0.53	0.71	0.84	0.63
**Load. item 4**	0.84	0.56	0.71	0.79
**Load. item 5**		0.51	0.56	0.82
**Load. item 6**		0.52	0.67	0.80
**Load. item 7**			0.75	0.65
**Load. item 8**			0.74	
**SS loadings**	**2.46**	**2.39**	**4.11**	**3.43**
**Proportion of var. explained**	**0.61**	**0.40**	**0.51**	**0.49**

Figures

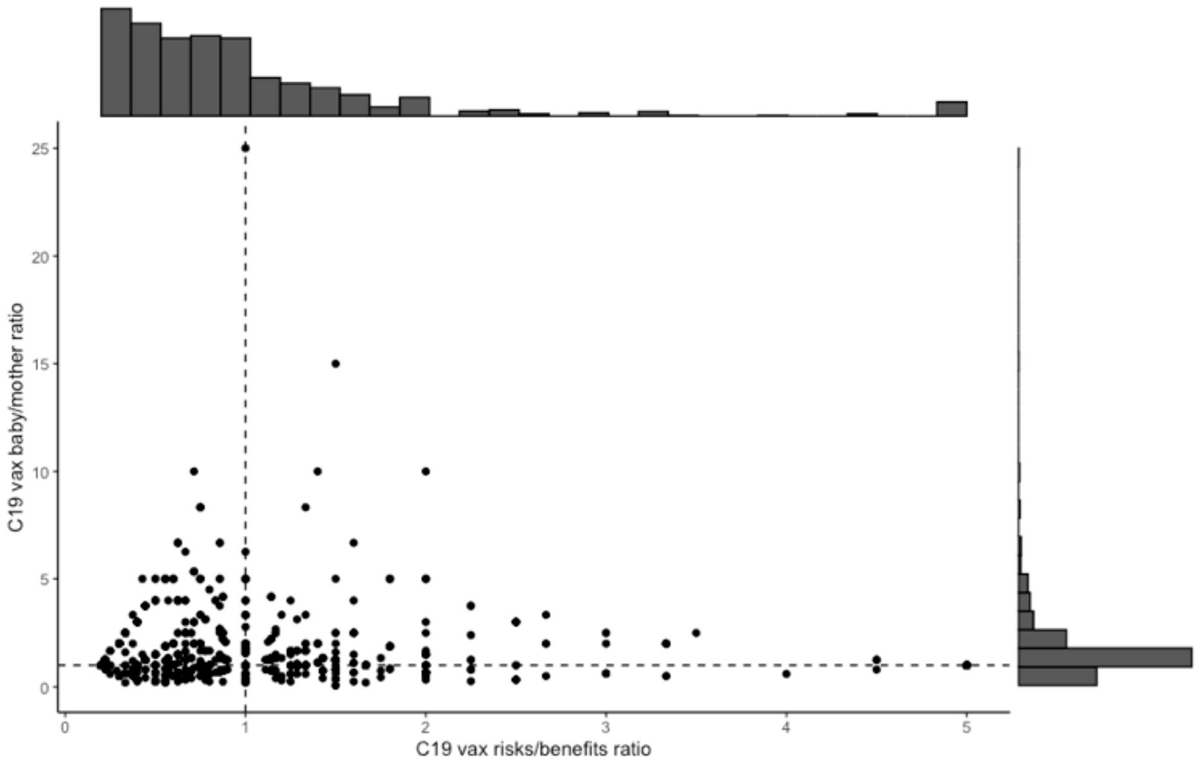

Figure S1. Joint and marginal distribution of the scores for C19 vax risks/benefits ratio and C19 vax baby/mother ratio. With the dotted lines the ratios equal to the unity.
